# Effects of a postpartum depression intervention: subgroup analyses from a cluster randomized trial

**DOI:** 10.3389/fpsyt.2026.1752138

**Published:** 2026-06-12

**Authors:** S. Darius Tandon, Alicia Diebold, Ann Kan, Jody Ciolino

**Affiliations:** 1 Department of Medical Social Sciences, Northwestern University Feinberg School of Medicine, Center for Community Health and, Chicago, IL, United States; 2 Department of Preventive Medicine, Northwestern University Feinberg School of Medicine, Chicago, IL, United States

**Keywords:** cognitive-behavioral therapy, home visiting, intervention, postpartum depression, prevention

## Abstract

**Introduction:**

Limited research has examined participant subgroups enrolled in postpartum depression interventions. Via a cluster-randomized trial of the Mothers and Babies (MB) postpartum depression preventive intervention, we examined changes in depressive symptoms across pre-specified participant subgroups: race/ethnicity, first-time mother, language of intervention receipt, and client education. We also examined interaction effects between subgroups and study arm (usual care vs. intervention) on symptom trajectories.

**Method:**

A total of 629 pregnant women (mean weeks’ gestation = 22.0 weeks, mean age 26.6 years) completed self-report assessments at baseline, post-intervention, and 12 and 24 weeks postpartum. The primary outcome was the Quick Inventory of Depressive Symptomatology (QIDS). Analyses involved linear mixed models fitted with baseline QIDS score, patient characteristics, study arm, and three-way interaction for QIDS scores at follow-up time points.

**Results:**

Minority participants exhibited larger mean QIDS decreases than non-minorities, with varying trajectories across study arm and minority subgroup (three-way interaction term *p* = 0.048). First-time mothers receiving MB also exhibited lower mean scores at 24 weeks postpartum than their multiparous counterparts over time (*p*=0.028). Trends in QIDS scores were comparable, although not significant, over time among women in the language (*p* = 0.929) and education subgroups (*p* = 0.635).

**Conclusions:**

MB may have greater benefits when delivered to racial and ethnic minorities and first-time mothers, providing added guidance to practitioners planning to implement the intervention. Future research should also consider additional organizational and system-level variables that may be associated with varied intervention effects.

## Introduction

Postpartum depression is a significant public health issue that is estimated to affect 10%–15% of women (1, 2). Among the factors most strongly associated with increased risk for postpartum depression are a history of depression, current depressive symptoms that do not reach diagnostic threshold, stressful life events, limited social and financial support, intimate partner violence, and low socioeconomic status (3, 4). Postpartum depression is associated with an array of adverse maternal and child health outcomes, including increased risk for suicidal behavior (5, 6). Of particular note is postpartum depression’s well-documented negative effects on parenting practices in which depressed mothers tend to be less spontaneous, responsive, and positive with their infants (7, 8). These suboptimal parenting practices, in turn, may lead to impaired parent–child attachment (9, 10) and infant social interaction difficulties and attachment insecurity (11–13). Longer-term effects of maternal depression are found as children move into early childhood and adolescence, with multiple longitudinal studies noting worse academic performance, increased rates of depression, and higher rates of internalizing and externalizing disorders among children and adolescents who lived with a depressed mother (14, 15).

Efficacious interventions that prevent the onset of postpartum depression and reduce depressive symptom severity exist (16–18). The United States Preventive Services Task Force (USPSTF) systematic review of postpartum depression preventive interventions found strong evidence that counseling interventions such as cognitive behavioral therapy and interpersonal therapy are effective in preventing postpartum depression (17). The USPSTF review on postpartum depression prevention noted the Mothers and Babies (MB) intervention as one of the two most efficacious counseling interventions, with multiple RCTs indicating MB’s efficacy when delivered in different settings and with women from multiple racial and ethnic groups (19–22). MB is a group-based intervention based on principles of cognitive behavioral therapy that promotes healthy mood management by teaching skills to increase the frequency of thoughts and behaviors that lead to positive mood states and help manage stress and depressive symptoms.

Across counseling interventions reviewed in the USPSTF report, the pooled relative risk (RR) reduction was 39%, with a pooled RR reduction across MB trials of 53% (17). These findings suggest that while counseling interventions, including MB, are efficacious compared to control groups, some women do not benefit as fully from receipt of these interventions. Said differently, improved outcomes associated with the receipt of a postpartum depression preventive intervention may not apply to all patient subgroups. Subgroup analyses are useful to generate more nuanced and precise statements about intervention effects across various patient characteristics. Moreover, subgroup analysis can provide intervention developers and practitioners who are interested in adopting an intervention with guidance on whether an intervention may need to be adapted in certain ways when delivered to certain participants. In fact, there is growing consensus that evidence-based interventions such as MB should consider adaptations to account for different organization, provider, and client-level characteristics (23) while ensuring that core elements of MB are delivered with high fidelity (24).

Unfortunately, previous postpartum depression prevention trials have largely lacked subgroup analyses. This is due, in part, to the size of the samples enrolled in these trials, which makes it difficult to generate stable estimates of intervention effects across subgroups. Examining previous MB trials helps illustrate sample size challenges associated with conducting subgroup analyses based on participant demographic characteristics. For example, in our MB trial conducted in Baltimore with women recruited from home visiting (HV) programs, over 80% of women in both the intervention and control groups were African-American, while more than 70% were first-time mothers (22). Another MB trial conducted in Hawaii with HV participants recruited >75% of women who identified as Asian/Pacific Islander (20).

The study upon which this manuscript is based was a cluster-randomized trial conducted with HV programs with a superiority aim comparing MB to usual HV and a non-inferiority aim comparing MB delivered by mental health professionals (MHP) versus MB delivered by lay home visitors (LHV). Additional details on our study protocol are found in Jensen et al. (25), and the results from our superiority and non-inferiority analyses are presented in Tandon et al. (26). A third aim of this study was to examine whether the effectiveness of the MB (usual care vs. intervention) varied according to key pre-specified patient characteristics. For this study’s aim, we used an as-treated analytic approach comparing intervention participants receiving a “full” intervention dose to controls. Specifically, we had an interest in the following patient characteristics: 1) race/ethnicity, 2) whether the participant was a first-time mother, 3) language of intervention receipt, and 4) education level. While other factors including, but not limited to, maternal age, personality traits, and social support may also affect intervention outcomes, these were selected *a priori* for analysis given their known variability in relation to postpartum depression (2, 3, 27), as well as variability among perinatal women enrolled in HV programs (28, 29)—the population for this cluster RCT.

This manuscript presents results examining whether and how the four pre-defined patient characteristics may be related to MB effectiveness. We first describe depressive symptom levels over time, for each pre-specified participant characteristic and study arm. Additionally, we examine the interaction between each participant characteristic and study arm (at least 4 MB LHV or MHP sessions vs. usual home visiting with 0 MB sessions) on depressive symptom trajectories.

## Materials and methods

### Study design

We conducted a secondary analysis of data from a cluster-randomized, non-inferiority study that randomized 45 HV programs to one of three arms using a 1:3:3 allocation ratio. For every one control program, we randomized three programs delivering MB via MHPs and three programs delivering MB via HVPs using covariate-constrained randomization techniques (30, 31) to control imbalance on three HV program-level variables: yearly client volume, population density of geographic area served, and percent minority clients. In the analyses, we adopted an “as-treated” approach: participants who did not receive any MB sessions, regardless of arm assignment, were considered as controls, and those who received at least 4+ MB sessions comprised the intervention arm. Any participant who received a “partial” dose of between 1 and 3 MB sessions was excluded from the analyses. Details on study design and randomization at the HV program (cluster) level are found elsewhere (25). While the unit of randomization was the HV program, the unit of analysis was the individual participant—i.e., we used the “cohort” study design for cluster-randomized trials as explained in Turner et al. (32). The design, hypotheses, and analysis plan of this study were preregistered.

### Study participants

HV programs recruited pregnant women enrolled at their agency, with some programs occasionally recruiting from their surrounding community. Potential participants were referred to the research team, who subsequently contacted participants to ensure that eligibility criteria were met—specifically, ≥16 years old, ≤33 weeks’ gestation upon referral, and Spanish- or English-speaking. Participants unreachable via phone, email, text, or social media received a recruitment letter in the mail. We received 1,316 referrals from participating HV programs, with 874 enrolling in the study (159 control, 310 MHP, 405 HVP). Our analytic sample size is 629, which includes all participants who received either no intervention or at least 4 MB sessions and contributed at least one follow-up assessment after baseline; 83 lost to follow-up participants and 162 participants who received a “partial dose” of intervention (between 1 and 3 MB sessions) were excluded from the analysis. No statistically significant demographic differences were found when comparing our full and analytic samples, nor were there differences between our analytic sample and those receiving a partial intervention dose (**Supplementary Table 1**).

Of the 629 participants included in this secondary data analysis, 271 (43.1%) received usual care and no intervention sessions, and 358 (56.9%) received at least 4+ MB sessions. Participants’ mean age was 26.6 ± 5.9 years and the median weeks’ gestation at baseline was 23 weeks (interquartile range: 16.5–28 weeks). Nearly 68% of the study participants self-identified as being a racial or ethnic minority (57.6% in usual care, 75.4% in intervention), 35.1% were first-time mothers (38.8% in usual care, 32.4% in intervention), 14.6% received the MB intervention in Spanish (10.0% in usual care, 18.2% intervention), and 58.7% had less than a college degree (61.6% in usual care, 56.4% in intervention). Across participants, 71.2% had a household income under $25K and 36.6% reported working part- or full-time. The mean baseline QIDS score is 7.97 ± 4.23 (7.63 ± 4.21 for usual care, 8.22 ± 4.23 for intervention). **Table 1** provides complete demographic summaries of study participants, with **Supplementary Table 2** also illustrating baseline QIDS severity levels.

**Table 1:** Demographic characteristics of the study participants (*N*=629).

Variable	Overall (*N*=629)	Control (*N*=271)	Intervention (*N*=358)
Age, mean (SD)	26.58 (5.92)	25.60 (5.71)	27.32 (5.97)
Weeks’ gestation at baseline, mean (SD)^a^	21.98 (7.08)	22.48 (7.24)	21.60 (6.94)
Employed, *n* (%)^b^	229 (36.64)	112 (41.79)	117 (32.77)
Income under $25K, *n* (%)^c^	437 (71.17)	176 (67.18)	261 (74.15)
Minority, *n* (%)	426 (67.73)	156 (57.56)	270 (75.42)
First-time mother, *n* (%)	221 (35.14)	105 (38.75)	116 (32.40)
Received intervention in Spanish, *n* (%)	92 (14.63)	27 (9.96)	65 (18.16)
Less than college education, *n* (%)	369 (58.66)	167 (61.62)	202 (56.42)
QIDS baseline score, mean (SD)	7.97 (4.23)	7.63 (4.21)	8.22 (4.23)

^a^ *N*=626.

^b^ *N*=625.

^c^ *N*=614.

### Intervention conditions

Participants in the MHP-led arm received MB delivered by an MHP trained on MB. MHPs were recruited from HV programs, or from state professional organizations when HV programs did not have staff meeting MHP facilitator criteria. An MHP was required to have at least 5 years’ experience working with families and children and at least a master’s degree in a mental health-related field. Participants in the HVP-led arm received MB led by an HVP employed at the participating HV program; we defined an HVP as someone with no more than a bachelor’s degree in child development, mental health, or a related field. The principal investigator (PI) led one- or one-and-a-half-day trainings with 53 individuals (32 HVPs, 21 MHPs) who delivered MB during this study. To promote intervention fidelity, facilitators received supervision from the PI while implementing their first cohort. Supervision involved weekly group calls with facilitators (HVP and MHP) to debrief the completed intervention session and plan for the subsequent session. Participants in the usual care arm received usual HV services but no MB intervention. Usual HV services were delivered in accordance with the HV model used by the program. In general, usual HV services focus on the preparation for childbirth and having a young child in the home, provision of emotional and tangible support, discussion of infant and young child development, and linkages to prenatal and pediatric care.

The MB group intervention consists of six sessions (i.e., one “cohort”). Sessions 1 and 2 introduce the importance of managing one’s mood and CBT content related to pleasant activities, including strategies for overcoming obstacles to engaging in pleasant activities by oneself and/or with others. Sessions 3 and 4 focus on identifying helpful and unhelpful thought patterns and introducing strategies for reframing unhelpful thoughts, while sessions 5 and 6 focus on promoting positive social interactions by helping clients expand their support networks, encouraging the use of assertive communication to meet one’s needs, and understanding how to manage stress associated with role changes of having a new child in the home.

Intervention sessions were delivered on a weekly basis, with some exceptions due to holidays or weather. One hundred thirty-two cohorts were delivered between January 2017 and October 2018 across programs randomized to either the MHP or HVP intervention arm. Sessions were held at the HV program or another community location on a day and time convenient for participants and facilitators. Cohorts had an average of three participants and were, on average, 86 min long.

### Data collection and instruments

All study activities were approved by the Northwestern University Institutional Review Board. Upon receiving a participant referral, a unique record was created in Research Electronic Data Capture (REDCap), a secure web application for managing online surveys and research data (33). Eligible and interested participants consented via REDCap, or by phone with a research assistant. All participants provided informed consent before baseline survey completion. Participant data were collected using self-report surveys at baseline, 1 week post-intervention (or 8 weeks post-enrollment for controls to align with the timing of intervention participants’ surveys), and 12 and 24 weeks postpartum. All data were collected via REDCap or by phone in English or Spanish. Data were collected from January 2017 to August 2019.

Depressive symptoms were assessed using the Quick Inventory of Depressive Symptomatology-Self-Report (QIDS-SR16). The QIDS-SR16 (34, 35) assessed the severity of depressive symptoms consistent with the Diagnostic and Statistical Manual (DSM-5) symptom criteria. Total scores range from 0 to 27; higher scores indicate greater symptomatology. QIDS-SR16 translates into depressive categories such that a score of 5 or less points indicates no depression, a score ranging from 6 to 10 indicates mild depression, 11–15 signifies moderate depression, 16–20 indicates severe depression, and anything above 20 would be labeled as very severe (IDS-QIDS).

### Data analysis

For our secondary analyses, we used an “as-treated” approach and excluded participants who received a “partial” dose. We also conducted sensitivity analyses using an intent-to-treat (ITT) approach, including all randomized participants and preserving random assignment, to assess the robustness of findings to varied intervention adherence.

The primary goal of this secondary data analysis was to evaluate whether the effectiveness of the intervention (usual care vs. MB) varies according to patient characteristics (e.g., minority, first-time mother, language of intervention receipt, education) and time. Participants were deemed minority based on self-reported race/ethnicity of either non-White and/or Hispanic ethnicity; a first-time mother was defined as having no previous children; language was categorized as either English or Spanish intervention receipt (or English/Spanish study assessments for those in the control study arm); education was categorized as at least some college or less than college education. Primary outcomes for analyses included the Quick Inventory of Depressive Symptomatology (QIDS) 16-item score at post intervention and 12 and 24 weeks postpartum.

Descriptive statistics summarized baseline characteristics at the participant level. Categorical variables were summarized with frequencies and percentages, and continuous variables were summarized with means and standard deviations or medians and interquartile ranges, as appropriate.

Primary analyses utilized separate linear mixed models (LMMs) to examine QIDS score by study arm, time point, and each participant-level characteristic of interest. That is, we explored each predictor (education, language, race, first-time mother) in separate models. For each of the four predictors, we compared all seven possible pairwise interactions between the study arm (A), predictor of interest (P), and time point (T) to the model with a three-way interaction term (A × C × T). We used the Akaike information criterion (AIC) to compare models with one another to determine the relative best-fitting model among all those examined. Ultimately, for each of the four variables, the three-way interaction term resulted in the best fit according to the AIC (**Supplementary Table 3a**). Specifically, the models we report hereafter for QIDS outcome include a fixed effect for the study arm, all four participant characteristics of interest, time point, the three-way interaction term (A × C × T), a random site intercept with variance components covariance structure to account for the same site within the study arm, and a random participant intercept with unstructured covariance structure to account for within-participant correlation. We also included fixed effects for pre-specified baseline variables thought to affect the outcome: baseline QIDS score, whether the participant met the criteria for a major depressive episode (MDE) at baseline, and whether they were on medication for depression or in counseling with a therapist at baseline as controlled factors. Forest plots displaying model-estimated QIDS score differences in the study arm, over time, were used to illustrate outcome differences across the relevant participant characteristics. These analyses were deemed exploratory or secondary to supplement the primary study findings. As such, we did not let statistical significance drive interpretation, but as above, we use the AIC as a relative model comparison. Bonferroni correction was applied to control the family-wise type 1 error rate to 5% for the analyses.

As supplemental, *post hoc* analyses, we explored the overlap between the subgroups of interest by conducting chi-squared tests to evaluate associations between all possible pairs of the four variables of primary interest (e.g., minority vs. first-time mom, minority vs. language, etc.). Since these tests resulted in several statistically significant associations, we created and summarized a new 16-strata (2 strata × 2 strata × 2 strata × 2 strata = 2^4^) variable to explore the overlap between these four variables overall and across the study arms (**Supplementary Tables 4**, **5**). We also included this new variable in the longitudinal model for QIDS, exploring the interaction between study arm, stratum, and time point. This model included a random participant effect and the same fixed effects as above, but due to stability issues, the random site effect was removed. Like the main models, the three-way interaction term resulted in the best fit according to the AIC (**Supplementary Table 3b**).

All analyses were conducted in SAS version 9.4 (The SAS Institute, Cary, NC) and the “forestplot” package (36) in R version 4.2.1 (37). All data, analysis code, and research materials are available upon reasonable request.

## Results

The best-fitting model according to the AIC for each of the participant-level characteristics of interest involved a three-way interaction term between characteristic, study arm, and time point. We summarize each of these model results, in turn, below.

**Figure 1:**
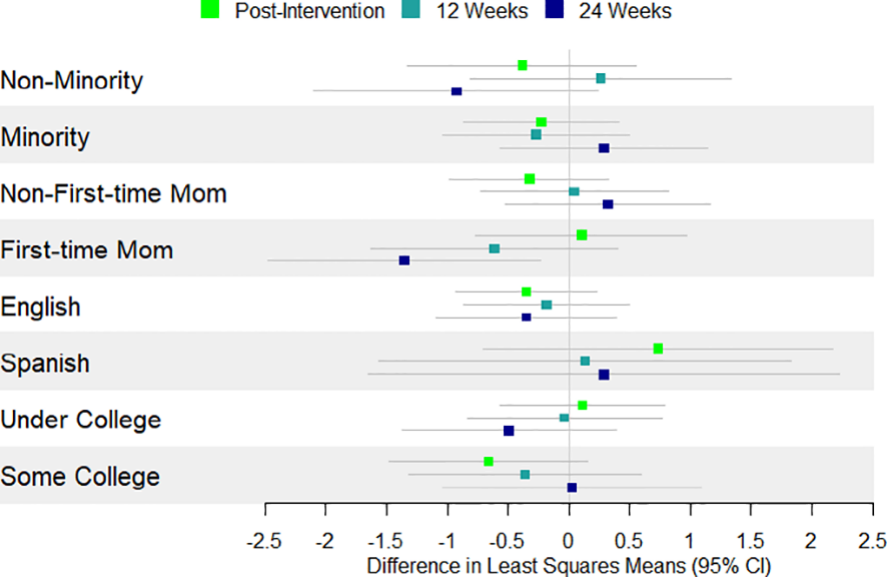
Model-estimated QIDS score difference in the intervention and control arms with 95% CI, by patient characteristics and time points. Forest plots displaying model-estimated Quick Inventory of Depressive Symptoms (QIDS) score differences in the intervention and control arms over time within each of the eight subgroups of interest: non-minority, minority, non-first-time mom, first-time mom, completed assessments in English, completed assessments in Spanish, had less than a college degree, and attended at least some college. Each level on the *x*-axis represents a subgroup of interest and presents the difference in model estimates between arms and their 95% confidence intervals at three follow-up time points: post-intervention, 12 weeks postpartum, and 24 weeks postpartum. The *y*-axis represents the intervention model-estimated QIDS score minus the control arm’s estimate. Since higher QIDS scores indicate more depressive symptoms, differences plotted to the left of the 0 line the intervention arm (the intervention arm showed lower QIDS scores than the control), and those plotted to the right of the 0 line favor the control arm (the intervention arm showed higher QIDS scores than the control).

When examining raw mean scores in QIDS within the minority vs. non-minority subgroups, we note similar patterns over time [minority—baseline: 8.13 (C) vs. 8.38 (I), 24 weeks: 5.71 vs. 5.73; non-minority—baseline: 6.96 vs. 7.72, 24 weeks: 6.26 vs. 5.79] (**Table 2**). In examining the model-estimated QIDS (three-way interaction term p = 0.048), participants self-identifying as belonging to a minority population tended to exhibit larger decreases in QIDS over time, with varying trajectories over time across the study arm and minority subgroup [minority—24 weeks minus post-intervention: −2.24 (C) vs. −1.72 (I); non-minority—24 weeks minus post-intervention: −0.74 (C) vs. −1.28 (I)] (**Table 3**). For between-study arm comparisons at the respective time points, refer to **Figure 1**.

**Table 2:** Mean QIDS score with standard deviation by study arm, patient characteristics, and time points.

Predictor level	Study arm	Baseline (*N*=629) *n*_c_ = 271, *n*_i_ = 358	Post-intervention (*N*=609) *n*_c_ = 258, *n*_i_ = 351	12 weeks (*N*=557) *n*_c_ = 234, *n*_i_ = 323	24 weeks (*N*=541) *n*_c_ = 226, *n*_i_ = 315		
Non-minority	Control	6.96 (3.64)	7.12 (3.54)	6.09 (4.29)		6.26 (4.65)
	Intervention	7.72 (4.2)	7.07 (3.92)	6.72 (4.7)		5.79 (4.47)
Minority	Control	8.13 (4.54)	7.41 (4.08)	5.71 (4.1)		5.43 (4.48)
	Intervention	8.38 (4.23)	7.38 (4.07)	5.73 (4.29)		5.55 (4.55)
Non-first-time mom	Control	7.22 (4.13)	7.15 (3.88)	5.9 (3.97)		5.49 (4.18)
	Intervention	7.85 (4.17)	7.04 (3.87)	6.08 (4.49)		5.79 (4.58)
First-time mom	Control	8.29 (4.27)	7.51 (3.81)	5.86 (4.51)		6.36 (5.14)
	Intervention	8.98 (4.27)	7.88 (4.31)	5.77 (4.27)		5.25 (4.41)
English	Control	7.84 (4.25)	7.57 (3.85)	6.12 (4.23)		6.02 (4.57)
	Intervention	8.69 (4.24)	7.64 (4.1)	6.42 (4.49)		5.96 (4.68)
Spanish	Control	5.77 (3.37)	4.85 (2.94)	3.88 (3.17)		3.81 (4.18)
	Intervention	6.07 (3.5)	5.83 (3.35)	4.11 (3.51)		4.1 (3.46)
< College education	Control	7.9 (4.46)	7.05 (3.7)	5.66 (4.19)		5.99 (4.85)
	Intervention	7.8 (4.09)	7.08 (4.04)	5.52 (4.35)		5.15 (4.38)
Some college	Control	7.2 (3.76)	7.63 (4.05)	6.21 (4.17)		5.56 (4.13)
	Intervention	8.76 (4.37)	7.59 (4)	6.56 (4.43)		6.2 (4.66)

n_c_, number of control arm participants who completed the QIDS assessment at the indicated time point; *n*_i_, number of intervention arm participants who completed the QIDS assessment at the indicated time point.

**Table 3:** Model-based estimated QIDS score with 95% CI by study arm, patient characteristics, and time points.

Predictor level	Study arm	Post-intervention	12 weeks	24 weeks
Non-minority	Control	8.16 [7.26, 9.05]	7.07 [6.11, 8.03]	7.42 [6.41, 8.43]
	Intervention	7.77 [6.84, 8.7]	7.33 [6.32, 8.33]	6.49 [5.42, 7.55]
Minority	Control	8.25 [7.43, 9.07]	6.69 [5.81, 7.57]	6.01 [5.08, 6.95]
	Intervention	8.02 [7.28, 8.75]	6.42 [5.64, 7.2]	6.3 [5.5, 7.11]
Non-first-time mom	Control	8.37 [7.57, 9.17]	7.14 [6.28, 8]	6.58 [5.68, 7.48]
	Intervention	8.04 [7.31, 8.78]	7.18 [6.41, 7.96]	6.9 [6.09, 7.71]
First-time mom	Control	8.08 [7.18, 8.98]	6.53 [5.56, 7.5]	6.94 [5.89, 7.98]
	Intervention	8.18 [7.32, 9.05]	5.91 [4.97, 6.86]	5.58 [4.59, 6.57]
English	Control	8.6 [7.89, 9.32]	7.22 [6.46, 7.97]	7.02 [6.23, 7.81]
	Intervention	8.25 [7.56, 8.94]	7.03 [6.31, 7.76]	6.67 [5.91, 7.42]
Spanish	Control	7.31 [5.95, 8.67]	6.23 [4.67, 7.79]	6.01 [4.24, 7.79]
	Intervention	8.05 [7.03, 9.06]	6.36 [5.24, 7.48]	6.3 [5.1, 7.5]
< College education	Control	7.92 [7.11, 8.73]	6.59 [5.72, 7.46]	6.7 [5.79, 7.62]
	Intervention	8.03 [7.26, 8.79]	6.55 [5.73, 7.37]	6.21 [5.36, 7.06]
Some college	Control	8.62 [7.74, 9.51]	7.23 [6.26, 8.2]	6.59 [5.55, 7.62]
	Intervention	7.96 [7.16, 8.76]	6.87 [6.01, 7.73]	6.61 [5.7, 7.52]

When examining raw mean scores in QIDS within the first-time mother versus non-first-time mother subgroups, we again note a similar trend over time [first-time mother—baseline: 8.29 (C) vs. 8.98 (I), 24 weeks: 6.36 (C) vs. 5.25 (I); non-first-time mother—baseline: 7.22 (C) vs. 7.85 (I), 24 weeks: 5.49 (C) vs. 5.79 (I)] (**Table 2**). In examining the model-estimated QIDS (three-way interaction term p = 0.028), we found that the intervention arm tended to have lower QIDS scores at 24 weeks postpartum in first-time mother compared to non-first-time mother [first-time mother—24 weeks: 6.94 (C) vs. 5.58 (I); non-first-time mother—24 weeks: 6.58 (C) vs. 6.90 (I)], suggesting a possible separation between the control and intervention study arms within the first-time mother subgroup (**Figure 1**), whereby the participants in the control arm tended to have higher QIDS scores 12 and 24 weeks postpartum. This pattern is not evident within the non-first-time mother subgroup (**Table 3**).

When examining raw mean scores in QIDS within the language subgroups, the Spanish-speaking participants tended to have lower QIDS scores overall at all time points in comparison to the English-speaking participants (**Table 2**). Overall, the trend over time for each of these subgroups across the study arms was comparable (three-way interaction *p*=0.929; **Table 3**). When examining QIDS over time within the education subgroups, we note similar overall findings, whereby changes over time across study arms were comparable (three-way interaction *p*=0.635; **Tables 2**, **3**).

In the ITT sensitivity analyses, none of the three-way interaction terms reached statistical significance, indicating attenuation of subgroup effects observed in the as-treated analysis (**Supplementary Table 6**). In the supplemental model containing the 16-strata variable, the three-way interaction term between the study arm, time point, and stratum was not statistically significant (*p* = 0.344).

## Discussion

Findings from the subgroup analyses of our cluster RCT suggest that MB may have greater benefits for women who belong to a minority racial/ethnic group and first-time mothers, while no differences were found in intervention effectiveness related to language of intervention receipt or education level. Our findings are significant based on research indicating that racial and ethnic minorities are more likely to experience postpartum depression relative to their non-minority counterparts (1, 2, 38). It also supports the results from previous RCTs that demonstrated MB’s effectiveness in reducing depressive symptoms among African-American (22), Latina (19, 21), and Native Hawaiian and Pacific Islanders (20). MB’s effectiveness among racial and ethnic minorities is also notable in relation to the ongoing implementation of MB in HV programs. The National Home Visiting Yearbook overview of HV client demographics across the United States indicates that 44% of HV clients from 17 evidence-based HV models are from a minority racial group, with 34% of women reporting Latina ethnicity (29). Given the limited uptake of mental health services among racial and ethnic minority HV clients referred to “traditional” mental health providers (39, 40), HV programs could benefit from interventions like MB that are integrated into the HV service model. It is also well-established that racial and ethnic minority women have less consistent access to high-quality mental health services, and even when services are available, there is often a lack of racially concordant care (41). Accordingly, it may be possible that the racial and ethnic minority women receiving MB were more invested in the intervention, given the lack of alternative mental health services and supports. Related to our results that MB may have greater benefits for first-time mothers, the transition to motherhood associated with the pregnancy of one’s first baby is a significant role change. This role change is associated with additional stress brought on by having a new child in the home, relationship conflict with one’s partner, and changing self-representation (4). These results, again, have implications for future work aimed at integrating interventions like MB into HV programs, as some HV models serve only first-time mothers, while others place increased emphasis on recruiting first-time mothers. While the MB curriculum explicitly discusses how pregnancy is a role transition for all individuals irrespective of whether one has previously given birth, our findings indicate that the skills provided in the curriculum related to navigating this role transition may be particularly helpful for first-time mothers.

When interpreting study results, caution should be used in generalizing findings to other perinatal populations engaged in HV given the considerable diversity among HV programs nationally. Four additional limitations are important to consider. First, our pre-specified demographic variables were limited to patient-level characteristics and did not examine potential HV program, provider, or system-level characteristics. For example, patient/provider concordance or provider cultural competency was not included as separate analytic variables. Additionally, HV programs vary in terms of the model used, with programs part of this study using five different models. For example, it is possible that some HV models have slightly more or less emphasis on mental health in their core curricula, or that home visitors from certain HV models receive more onboarding related to addressing client mental health, which could lead to their greater reinforcement of MB skills during their home visits with clients. It is also possible that other patient-level variables such as maternal age, attachment style, or perceived social support could also have influenced our primary outcome. Second, our analyses were conducted using a derived exposure variable to examine the relationship between individuals deemed to receive a “full dose” of the intervention, which we have defined in previous studies as receiving four or more of the six MB sessions. We felt that this analytic approach best provided data related to our question of for whom the MB intervention appears to be working best, as we excluded individuals who had less engagement with the intervention. The absence of statistically significant subgroup effects in the ITT analysis suggests that subgroup differences reflect the effects of intervention exposure rather than assignment alone. An important future direction, therefore, would be to determine strategies to promote greater intervention attendance. Third, because our criteria for study enrollment were based on postpartum depression risk factors other than depressive symptomatology score, we recruited some women with minimal baseline symptom levels. This created a flooring effect in which there was less room to demonstrate symptom reduction, which may contribute to our non-significant findings. Finally, we also note that while treatment fidelity could also influence outcomes, we did not examine the potential moderating effects of intervention fidelity on study outcomes.

Further research is needed to understand the potential differential effects of postpartum depression preventive interventions across participant subgroups. Farrell et al. (42) note that this research should be theoretically grounded rather than relying on *post hoc* analyses that examine several potential moderating variables. We propose that researchers consider juxtaposing theoretically derived variables with pragmatic variables that help practitioners understand the potential impact of evidence-based interventions across the populations with whom they work. As this relates to the current study, home visiting partners with whom we collaborated were keenly interested in understanding whether MB’s effectiveness varied across the four patient characteristics—race/ethnicity, first-time mother, language of intervention receipt, and education level—to help guide their decisions about MB’s appropriateness for the patient population they serve. This study, and others similarly looking at subgroup differences related to postpartum depression interventions, can also help identify areas where model developers and practitioners may need to consider fidelity-consistent adaptations (43) to intervention content and delivery methods to ensure interventions meet the needs of the diversity of intervention recipients.

This study represents one of the first examinations of subgroup differences associated with a postpartum depression preventive intervention. While examining subgroup differences in intervention outcomes has long been recognized as an important undertaking, there is still considerable variability in whether behavioral intervention trials examine and report on these differences. This is true despite increasing calls by groups such as the Cochrane Collaborative to consider demographic subgroups in the design and interpretation of clinical trials (44). Results of subgroup analyses are consistent with precision medicine that focuses on differentiating what intervention works for whom and under what conditions (45). By ascertaining how interventions affect across patient subgroups, researchers and policymakers gain valuable insights that can help guide intervention refinement to meet the needs of individuals who benefit less from an existing intervention.

## Data Availability

The raw data supporting the conclusions of this article will be made available by the authors, without undue reservation.
