# Supplementary material for: Effects of a postpartum depression intervention: subgroup analyses from a cluster randomized trial
Source: Front Psychiatry. 2026 Jun 12;17:1752138. doi: 10.3389/fpsyt.2026.1752138 (PMC13307506; doi:10.3389/fpsyt.2026.1752138)
Supplement: Supplementary file 1 [file Table1.docx]

|  | **Overall Sample** | **Analytic**  **Sample** | **Lost to**  **follow-up** | **Partial Intervention** |
| --- | --- | --- | --- | --- |
| Overall, n (%) | **874** | **629 (72.0%)** | **83 (9.5%)** | **162 (18.5%)** |
| Age, mean (SD) | 26.09 (5.81) | 26.58 (5.92) | 23.44 (5.32) | 25.55 (5.25) |
| Weeks’ gestation at baseline, mean (SD)* | 22.15 (7.38) | 21.98 (7.08) | 19.81 (7.79) | 23.79 (8.00) |
| Employed, n (%)** | 305 (35.76%) | 229 (36.64%) | 23 (33.82%) | 53 (33.13%) |
| Income under $25K, n (%) ^ | 612 (73.56%) | 437 (71.17%) | 44 (72.13%) | 131 (83.44%) |
| Minority, n (%) | 617 (70.59%) | 426 (67.73%) | 64 (77.11%) | 127 (78.40%) |
| First-time mother, n (%) | 319 (36.50%) | 221 (35.14%) | 34 (40.96%) | 64 (39.51%) |
| Spanish intervention recipient, n (%) | 113 (12.93%) | 92 (14.63%) | 4 (4.82%) | 17 (10.49%) |
| Less than college education, n (%) | 531 (60.76%) | 369 (58.66%) | 65 (78.31%) | 97 (59.88%) |
| QIDS baseline score, mean (SD) ^^ | 7.95 (4.24) | 7.97 (4.23) | 8.18 (4.51) | 7.79 (4.16) |

**Supplemental Table 1.**

*Demographics by Analytic Group.*

*N=854. **N=853. ^N=832. ^^N=859.
