# Supplementary material for: Effects of a postpartum depression intervention: subgroup analyses from a cluster randomized trial
Source: Front Psychiatry. 2026 Jun 12;17:1752138. doi: 10.3389/fpsyt.2026.1752138 (PMC13307506; doi:10.3389/fpsyt.2026.1752138)
Supplement: Supplementary file 2 [file Table2.docx]

**Supplemental Table 2**

1. *Frequency of mothers with no, mild, or moderate and above QIDS score at Baseline, by study arm.*

| N (%) | **Overall, N=629** | **Control,**  **N=271** | **Intervention,**  **N=358** |
| --- | --- | --- | --- |
| **QIDS Score at Baseline** |  |  |  |
| None (0-5) | 216 (34.3%) | 105 (38.8%) | 111 (31.0%) |
| Mild (6-10) | 252 (40.1%) | 104 (38.4%) | 148 (41.3%) |
| Moderate and Above (11+) | 161 (25.6%) | 62 (22.9%) | 99 (27.7%) |

1. *Frequency of mothers with no, mild, or moderate and above QIDS score at baseline by subgroup (minority, education level, language of intervention receipt, and new mom).*

| N (%) | **Overall, N=629** | **Under College,  N=369** | **Some College,  N=260** |
| --- | --- | --- | --- |
| **QIDS Score at Baseline** |  |  |  |
| None (0-5) | 216 (34.3%) | 130 (35.2%) | 86 (33.1%) |
| Mild (6-10) | 252 (40.1%) | 145 (39.3%) | 107 (41.2%) |
| Moderate and Above (11+) | 161 (25.6%) | 94 (25.5%) | 67 (25.8%) |

| N (%) | **Overall,**  **N=629** | **White/Caucasian,**  **N=203** | **Minority,**  **N=426** |
| --- | --- | --- | --- |
| **QIDS Score at Baseline** |  |  |  |
| None (0-5) | 216 (34.3%) | 78 (38.4%) | 138 (32.4%) |
| Mild (6-10) | 252 (40.1%) | 84 (41.4%) | 168 (39.4%) |
| Moderate and Above (11+) | 161 (25.6%) | 41 (20.2%) | 120 (28.2%) |

| N (%) | **Overall,**  **N=629** | **English,**  **N=537** | **Spanish,**  **N=92** |
| --- | --- | --- | --- |
| **QIDS Score at Baseline** |  |  |  |
| None (0-5) | 216 (34.3%) | 159 (29.6%) | 57 (62.0%) |
| Mild (6-10) | 252 (40.1%) | 226 (42.1%) | 26 (28.3%) |
| Moderate and Above (11+) | 161 (25.6%) | 152 (28.3%) | 9 (9.8%) |

| N (%) | **Overall,**  **N=629** | **Non-First-time Mom,**  **N=408** | **First-time Mom,**  **N=221** |
| --- | --- | --- | --- |
| **QIDS Score at Baseline** |  |  |  |
| None (0-5) | 216 (34.3%) | 155 (38.0%) | 61 (27.6%) |
| Mild (6-10) | 252 (40.1%) | 158 (38.7%) | 94 (42.5%) |
| Moderate and Above (11+) | 161 (25.6%) | 95 (23.3%) | 66 (29.9%) |
