# Supplementary material for: Effects of a postpartum depression intervention: subgroup analyses from a cluster randomized trial
Source: Front Psychiatry. 2026 Jun 12;17:1752138. doi: 10.3389/fpsyt.2026.1752138 (PMC13307506; doi:10.3389/fpsyt.2026.1752138)
Supplement: Supplementary file 3 [file Table3.docx]

**Supplemental Table 3**

*Summary of all models explored and their respective AICs.*

**3a. Main analysis**

| **Primary predictor of interest** | **Model** | **AIC** |
| --- | --- | --- |
| Education | timept*predictor*sessions_4plus | 8847.3 |
|  | predictor*sessions_4plus | 8851.4 |
|  | time*predictor predictor*sessions_4plus | 8851.6 |
|  | time*sessions_4plus predictor*sessions_4plus | 8852.3 |
|  | time*predictor time*sessions_4plus predictor*sessions_4plus | 8852.5 |
|  | time*predictor | 8852.8 |
|  | time*sessions_4plus | 8853.5 |
|  | time*predictor time*sessions_4plus | 8853.7 |
| First-Time Mom | timept*predictor*sessions_4plus | 8836.4 |
|  | time*predictor predictor*sessions_4plus | 8845.4 |
|  | time*predictor | 8845.9 |
|  | time*predictor time*sessions_4plus predictor*sessions_4plus | 8846.3 |
|  | time*predictor time*sessions_4plus | 8846.8 |
|  | predictor*sessions_4plus | 8852.1 |
|  | time*sessions_4plus predictor*sessions_4plus | 8853.0 |
|  | time*sessions_4plus | 8853.5 |
| Language | timept*predictor*sessions_4plus | 8846.3 |
|  | time*predictor predictor*sessions_4plus | 8849.5 |
|  | predictor*sessions_4plus | 8850.1 |
|  | time*predictor time*sessions_4plus predictor*sessions_4plus | 8850.3 |
|  | time*sessions_4plus predictor*sessions_4plus | 8851.0 |
|  | time*predictor | 8852.0 |
|  | time*predictor time*sessions_4plus | 8852.8 |
|  | time*sessions_4plus | 8853.5 |
| Minority | timept*predictor*sessions_4plus | 8837.7 |
|  | time*predictor predictor*sessions_4plus | 8845.9 |
|  | time*predictor | 8846.3 |
|  | time*predictor time*sessions_4plus predictor*sessions_4plus | 8846.4 |
|  | time*predictor time*sessions_4plus | 8846.9 |
|  | predictor*sessions_4plus | 8852.1 |
|  | time*sessions_4plus predictor*sessions_4plus | 8853.0 |
|  | time*sessions_4plus | 8853.5 |

**3b. Post-hoc analyses**

| **Primary Predictor of Interest** | **Interaction Term in Model** | **AIC** |
| --- | --- | --- |
| 16- strata variable “stratum” | timept*stratum*sessions_4plus | 8706.8 |
|  | timept*stratum timept*sessions_4plus stratum*sessions_4plus | 8797.4 |
|  | timept*stratum stratum*sessions_4plus | 8796.9 |
|  | timept*sessions_4plus stratum*sessions_4plus | 8855.8 |
|  | stratum*sessions_4plus | 8854.9 |
|  | timept*stratum | 8823.6 |
|  | timept*stratum timept*sessions_4plus | 8824.1 |
|  | timept*sessions_4plus | 8882.2 |
