# Supplementary material for: Effects of a postpartum depression intervention: subgroup analyses from a cluster randomized trial
Source: Front Psychiatry. 2026 Jun 12;17:1752138. doi: 10.3389/fpsyt.2026.1752138 (PMC13307506; doi:10.3389/fpsyt.2026.1752138)
Supplement: Supplementary file 4 [file Table4.docx]

**Supplemental Table 4**

*Sixteen strata created with the 4 variables (N, %) overall and by study arm.*

| **Stratum** | **Overall,**  **N=629** | **Control,**  **N=271** | **Intervention,**  **N=358** |
| --- | --- | --- | --- |
| Minority, English, Under College, Non-New Mom | 129 (20.5%) | 56 (20.7%) | 73 (20.4%) |
| Minority, English, Some College, Non-New Mom | 86 (13.7%) | 26 (9.6%) | 60 (16.8%) |
| Minority, English, Under College, New Mom | 76 (12.1%) | 35 (12.9%) | 41 (11.5%) |
| Non-Minority, English, Some College, Non-New Mom | 68 (10.8%) | 42 (15.5%) | 26 (7.3%) |
| Non-Minority, English, Under College, Non-New Mom | 54 (8.6%) | 27 (10.0%) | 27 (7.5%) |
| Minority, Spanish, Under College, Non-New Mom | 51 (8.1%) | 14 (5.2%) | 37 (10.3%) |
| Non-Minority, English, Under College, New Mom | 46 (7.3%) | 28 (10.3%) | 18 (5.0%) |
| Minority, English, Some College, New Mom | 44 (7.0%) | 12 (4.4%) | 32 (8.9%) |
| Non-Minority, English, Some College, New Mom | 34 (5.4%) | 18 (6.6%) | 16 (4.5%) |
| Minority, Spanish, Some College, Non-New Mom | 19 (3.0%) | 1 (0.4%) | 18 (5.0%) |
| Minority, Spanish, Under College, New Mom | 12 (1.9%) | 7 (2.6%) | 5 (1.4%) |
| Minority, Spanish, Some College, New Mom | 9 (1.4%) | 5 (1.9%) | 4 (1.1%) |
| Non-Minority, Spanish, Under College, Non-New Mom | 1 (0.2%) | 0 (0.0%) | 1 (0.3%) |
| Non-Minority, Spanish, Some College, New Mom | 0 (0%) | 0 (0%) | 0 (0%) |
| Non-Minority, Spanish, Some College, Non-New Mom | 0 (0%) | 0 (0%) | 0 (0%) |
| Non-Minority, Spanish, Under College, New Mom | 0 (0%) | 0 (0%) | 0 (0%) |
