# Supplementary material for: Effects of a postpartum depression intervention: subgroup analyses from a cluster randomized trial
Source: Front Psychiatry. 2026 Jun 12;17:1752138. doi: 10.3389/fpsyt.2026.1752138 (PMC13307506; doi:10.3389/fpsyt.2026.1752138)
Supplement: Supplementary file 5 [file Table5.docx]

**Supplemental Table 5**

*QIDS score with 95% CI at 24 weeks, estimated by model containing 16-level strata variable.*

| **Stratum** | **Study Arm** | **Timepoint** | **Estimate [95% CI]** |
| --- | --- | --- | --- |
| Non-Minority, English, Under College, Non-New Mom | Intervention | 24 Weeks | 6.97 [5.53, 8.41] |
| Non-Minority, English, Under College, New Mom | Intervention | 24 Weeks | 6.73 [4.91, 8.55] |
| Non-Minority, English, Some College, Non-New Mom | Intervention | 24 Weeks | 7.33 [5.84, 8.83] |
| Non-Minority, English, Some College, New Mom | Intervention | 24 Weeks | 5.81 [3.96, 7.66] |
| Non-Minority, Spanish, Under College, Non-New Mom | Intervention | 24 Weeks | 2.55 [-4.32, 9.42] |
| Minority, English, Under College, Non-New Mom | Intervention | 24 Weeks | 6.41 [5.35, 7.47] |
| Minority, English, Under College, New Mom | Intervention | 24 Weeks | 5.34 [4.02, 6.66] |
| Minority, English, Some College, Non-New Mom | Intervention | 24 Weeks | 7.68 [6.55, 8.81] |
| Minority, English, Some College, New Mom | Intervention | 24 Weeks | 5.63 [4.23, 7.02] |
| Minority, Spanish, Under College, Non-New Mom | Intervention | 24 Weeks | 6.67 [5.34, 7.99] |
| Minority, Spanish, Under College, New Mom | Intervention | 24 Weeks | 4.70 [1.57, 7.83] |
| Minority, Spanish, Some College, Non-New Mom | Intervention | 24 Weeks | 6.01 [4.20, 7.82] |
| Minority, Spanish, Some College, New Mom | Intervention | 24 Weeks | 5.43 [1.96, 8.90] |
| Non-Minority, English, Under College, Non-New Mom | Control | 24 Weeks | 8.07 [6.54, 9.61] |
| Non-Minority, English, Under College, New Mom | Control | 24 Weeks | 8.39 [6.91, 9.87] |
| Non-Minority, English, Some College, Non-New Mom | Control | 24 Weeks | 7.03 [5.81, 8.24] |
| Non-Minority, English, Some College, New Mom | Control | 24 Weeks | 8.11 [6.41, 9.81] |
| Minority, English, Under College, Non-New Mom | Control | 24 Weeks | 6.13 [4.97, 7.29] |
| Minority, English, Under College, New Mom | Control | 24 Weeks | 6.71 [5.23, 8.19] |
| Minority, English, Some College, Non-New Mom | Control | 24 Weeks | 6.72 [5.13, 8.30] |
| Minority, English, Some College, New Mom | Control | 24 Weeks | 4.43 [2.10, 6.76] |
| Minority, Spanish, Under College, Non-New Mom | Control | 24 Weeks | 6.13 [4.14, 8.12] |
| Minority, Spanish, Under College, New Mom | Control | 24 Weeks | 3.49 [0.18, 6.79] |
| Minority, Spanish, Some College, Non-New Mom | Control | 24 Weeks | 2.55 [-4.32, 9.42] |
| Minority, Spanish, Some College, New Mom | Control | 24 Weeks | 8.40 [4.55, 12.25] |
