# Supplementary material for: Effects of a postpartum depression intervention: subgroup analyses from a cluster randomized trial
Source: Front Psychiatry. 2026 Jun 12;17:1752138. doi: 10.3389/fpsyt.2026.1752138 (PMC13307506; doi:10.3389/fpsyt.2026.1752138)
Supplement: Supplementary file 6 [file Table6.docx]

**Supplemental Table 6**

*Intention-to-treat (ITT) Model based estimated QIDS score with 95% CI by study arm, patient characteristics and time points.*

| **Predictor Level** | **Study Arm** | **Post-Intervention** | **12 Weeks** | **24 Weeks** |
| --- | --- | --- | --- | --- |
| Non-Minority | Control | 8.51 [7.65,9.38] | 7.47 [6.51,8.43] | 7.55 [6.56,8.54] |
|  | Intervention | 7.86 [7.09,8.62] | 7.24 [6.41,8.07] | 6.96 [6.11,7.8] |
| Minority | Control | 9.37 [8.35,10.39] | 7.29 [6.08,8.49] | 6.73 [5.48,7.99] |
|  | Intervention | 8.13 [7.49,8.77] | 6.62 [5.95,7.29] | 6.16 [5.48,6.84] |
| Non-First-time Mom | Control | 8.78 [7.91,9.64] | 7.27 [6.29,8.24] | 6.97 [5.98,7.97] |
|  | Intervention | 8.09 [7.45,8.72] | 7.13 [6.46,7.79] | 6.68 [6,7.36] |
| First-time Mom | Control | 8.89 [7.89,9.9] | 7.66 [6.52,8.8] | 7.85 [6.63,9.08] |
|  | Intervention | 8.18 [7.44,8.91] | 6.25 [5.46,7.03] | 5.9 [5.1,6.7] |
| English | Control | 9.28 [8.52,10.04] | 7.84 [6.99,8.69] | 7.63 [6.76,8.5] |
|  | Intervention | 8.33 [7.74,8.92] | 7.07 [6.45,7.68] | 6.62 [6,7.25] |
| Spanish | Control | 7.29 [5.66,8.93] | 6.19 [4.25,8.12] | 6.79 [4.56,9.02] |
|  | Intervention | 8.09 [7.22,8.95] | 6.58 [5.62,7.55] | 6.31 [5.31,7.31] |
| < College Education | Control | 8.73 [7.83,9.64] | 7.45 [6.42,8.47] | 7.52 [6.44,8.6] |
|  | Intervention | 7.96 [7.29,8.62] | 6.48 [5.78,7.18] | 6.26 [5.56,6.97] |
| Some College | Control | 8.89 [7.95,9.82] | 7.36 [6.29,8.43] | 7.03 [5.93,8.14] |
|  | Intervention | 8.25 [7.56,8.94] | 7.21 [6.47,7.94] | 6.5 [5.74,7.25] |
